# A retrospective study of HIV pre‐exposure prophylaxis counselling among non‐Hispanic Black youth diagnosed with bacterial sexually transmitted infections in the United States, 2014–2019

**DOI:** 10.1002/jia2.25867

**Published:** 2022-02-22

**Authors:** Dovie L. Watson, Pamela A. Shaw, Danielle T. Petsis, Julia Pickel, José A. Bauermeister, Ian Frank, Sarah M. Wood, Robert Gross

**Affiliations:** ^1^ Department of Medicine (Infectious Diseases) University of Pennsylvania Perelman School of Medicine Philadelphia Pennsylvania USA; ^2^ Department of Biostatistics Epidemiology and Informatics University of Pennsylvania Perelman School of Medicine Philadelphia Pennsylvania USA; ^3^ Kaiser Permanente Washington Health Research Institute Seattle Washington USA; ^4^ Craig Dalsimer Division of Adolescent Medicine Children's Hospital of Philadelphia Philadelphia Pennsylvania USA; ^5^ PolicyLab Children's Hospital of Philadelphia Philadelphia Pennsylvania USA; ^6^ Department of Family & Community Health University of Pennsylvania Philadelphia Pennsylvania USA; ^7^ Department of Pediatrics University of Pennsylvania Perelman School of Medicine Philadelphia Pennsylvania USA

**Keywords:** pre‐exposure prophylaxis, adolescents, sexually transmitted diseases, key and vulnerable populations, HIV prevention, gender

## Abstract

**Introduction:**

Youth account for a disproportionate number of new HIV infections; however, pre‐exposure prophylaxis (PrEP) use is limited. We evaluated PrEP counselling rates among non‐Hispanic Black youth in the United States after a bacterial sexually transmitted infection (STI) diagnosis.

**Methods:**

We conducted a retrospective cohort study of Black youth receiving care at two academically affiliated clinics in Philadelphia between June 2014 and June 2019. We compared PrEP counselling for youth who received primary care services versus those who did not receive primary care services, all of whom met PrEP eligibility criteria due to STI diagnosis per U.S. Centers for Disease Control and Prevention clinical practice guidelines. Two logistic regression models for receipt of PrEP counselling were fit: Model 1 focused on sexual and gender minority (SGM) status and Model 2 on rectal STIs with both models adjusted for patient‐ and healthcare‐level factors.

**Results:**

Four hundred and sixteen patients met PrEP eligibility criteria due to STI based on sex assigned at birth and sexual partners. Thirty patients (7%) had documentation of PrEP counselling. Receipt of primary care services was not significantly associated with receipt of PrEP counselling in either Model 1 (adjusted OR (aOR) 0.10 [95% CI 0.01, 0.99]) or Model 2 (aOR 0.52 [95% CI 0.10, 2.77]). Receipt of PrEP counselling was significantly associated with later calendar years of STI diagnosis (aOR 6.80 [95% CI 1.64, 29.3]), assigned male sex at birth (aOR 26.2 [95% CI 3.46, 198]) and SGM identity (aOR 317 [95% CI 39.9, 2521]) in Model 1 and later calendar years of diagnosis (aOR 3.46 [95% CI 1.25, 9.58]), assigned male sex at birth (aOR 18.6 [95% CI 3.88, 89.3]) and rectal STI diagnosis (aOR 28.0 [95% CI 8.07, 97.5]) in Model 2. Fourteen patients (3%) started PrEP during the observation period; 12/14 (86%) were SGM primary care patients assigned male sex at birth.

**Conclusions:**

PrEP counselling and uptake among U.S. non‐Hispanic Black youth remain disproportionately low despite recent STI diagnosis. These findings support the need for robust investment in PrEP‐inclusive sexual health services that are widely implemented and culturally tailored to Black youth, particularly cisgender heterosexual females.

## 1 INTRODUCTION

Human immunodeficiency virus (HIV) and sexually transmitted infections (STIs) among youth (individuals aged 13–24 years) are an enduring public health issue in the United States. In 2018, the Centers for Disease Control and Prevention (CDC) estimated 21% of 37,832 new HIV diagnoses were among youth—approximately 50% of whom were non‐Hispanic Black youth [1, 2]. Youth accounted for half of 19 million incident STI diagnoses in 2018, with non‐Hispanic Black youth disproportionately affected [3]. In 2012, the U.S. Food and Drug Administration (FDA) approved emtricitabine (FTC)/tenofovir disoproxil fumarate (TDF)—an antiretroviral medication previously approved for HIV treatment in adults and adolescents [4, 5]—for pre‐exposure prophylaxis (PrEP) in adults at increased risk for HIV acquisition and expanded PrEP approval to include adolescents under 18 years of age in 2018 [6, 7, 8]. Per CDC guidelines, a bacterial STI diagnosis in the previous 6 months was an indication due to sexual risk behaviour [6, 9]. Although youth account for a disproportionate number of incident HIV and bacterial STI infections, PrEP use among U.S. youth remains limited [7, 10, 11, 12, 13, 14, 15, 16].

Youth experience unique barriers to sexual health services beyond those known to undermine linkage to PrEP services among other key populations (e.g. lack of provider knowledge and willingness to prescribe PrEP, clinic logistics, stigmatizing provider behaviour and decision‐making biases, and lack of accessible PrEP providers) [17, 18, 19, 20, 21, 22, 23, 24, 25]. Interpersonal dynamics within the provider–patient dyad may affect linkage to PrEP care among youth in ways that are challenging to identify [14, 25]. Literature has been mixed regarding the influence of having a primary care provider on receipt of sexual health services among youth. Prior studies have found that when young people have established relationships with a trusted healthcare provider and confidentiality concerns are addressed, they are more likely to access healthcare, communicate about sexual and mental health, and return for care [26, 27, 28, 29, 30, 31, 32]. Conversely, studies have demonstrated suboptimal rates of HIV/STI screening and sexual health‐related discussions between youth and healthcare providers [30, 31, 32, 33, 34, 35, 36], which may adversely affect the provider's ability to assess the patient's sexual health goals, HIV risk and prevention behaviours, and HIV prevention needs—including provider‐initiated PrEP counselling with PrEP‐eligible youth.

PrEP‐related expenses (e.g. medication costs, appointments, HIV/STI testing and monitoring labs), dependence on parent/guardian insurance, concerns about inadvertent disclosure of sexual risk behaviours and lack of clarity regarding parental consent requirements further complicate PrEP access for youth [3, 14, 25, 37, 38, 39, 40, 41, 42, 43, 44, 45]. Many public/government health insurance plans (e.g. Pennsylvania Medicaid/Children's Health Insurance Program) and commercial plans cover these expenses, but they often require prior authorization from the primary account holder (e.g. parent) or cost‐prohibitive co‐pays [46, 47, 48, 49]. Youth who are uninsured or do not wish to use parental insurance might use the medication assistance program offered by the manufacturer to cover the cost of the medication. However, one must be 18 years or older to be eligible for this program [25, 47], and out‐of‐pocket cost for appointments and tests may also be unaffordable [12].

The ways in which these complex and interrelated factors impact a young person's opportunities for PrEP counselling during both primary care and sexual health visits remain unclear. Our primary objective was to examine PrEP counselling rates among U.S. non‐Hispanic Black youth after an incident bacterial STI diagnosis at combined primary care/government subsidized (i.e. Title X) clinics and identify patient‐ and healthcare‐level factors related to receipt of PrEP counselling. We hypothesized that among a cohort comprised of non‐Hispanic Black youth at higher risk for HIV acquisition due to incident bacterial STI diagnosis, primary care providers would recognize STI diagnosis as an opportunity to provide PrEP counselling, and receipt of primary care services would be positively associated with receipt of PrEP counselling.

## 2 METHODS

### 2.1 Study design

We conducted a retrospective cohort study of a convenience sample of youth aged 13–24 years who sought STI testing and treatment between June 2014 and June 2019 at two academically affiliated clinics that both provided collocated paediatric/adolescent primary care services and confidential sexual/reproductive health services in Philadelphia, Pennsylvania [36, 50]. Youth without insurance were not able to access primary care services at the sites. The services provided at each of the two clinics were identical in scope. Confidential sexual and reproductive health services were provided to youth regardless of insurance status using U.S. Federal Title X‐grant Family Planning funding. At both clinics, providers were able to document Title X notes with additional billing and electronic health record (EHR) privacy protections to mitigate the risk of disclosure from documentation. STI testing was standard practice at these sites for youth who (1) reported symptoms or a partner with an STI, (2) requested STI testing, (3) reported inconsistent condom use during visit (e.g. visit for primary care services; visit for contraceptive services) or (4) met criteria for annual chlamydia and/or gonorrhoea screening [51, 52] regardless of reason for visit. Both clinics were comprised of a multidisciplinary team of providers (e.g. certified registered nurse practitioners, residents, adolescent medicine fellows, paediatric and adolescent medicine attendings) affiliated with the same academic department. Providers at both clinics were well‐versed with Pennsylvania statues regarding “mature minors” (i.e. those independently seeking sexual health services) and routinely provided sexual health‐related services without parental consent. Given safety data in the adolescent population from HIV treatment trials, the clear and pressing need for prevention, lack of explicit age cutoff in the FDA indication [5], limited but observable prescribing of PrEP to minors nationally [7, 8, 13, 47, 53, 54, 55], department leadership concluded that prescribing PrEP to “mature minors” without parental consent was in accordance with Pennsylvania state licensure. Clinical trainings on PrEP prescribing were provided at both clinics, which included the importance of PrEP for minor adolescents at increased risk for HIV infection. We compared the documentation of PrEP counselling in narrative clinic notes by primary care status. Of note, PrEP counselling was not prompted in the EHR.

Patients at both sites were mostly African American (87%) and Medicaid insured (79%). Patients were included if they were aged 13–24 years at the time of STI diagnosis, non‐Hispanic Black/African American and had a qualifying STI diagnosis for PrEP counselling based on sex assigned at birth and self‐reported sex of sexual partners [6, 9]. Patients were excluded if they had a previously documented PrEP prescription at the time of first qualifying STI diagnosis or known HIV‐positive status prior to first qualifying STI diagnosis during the study period. The “Sexually Transmitted Infections Among Adolescents in Primary Care Settings in Philadelphia” study protocol was reviewed by the Institutional Review Boards (IRBs) at the Children's Hospital of Philadelphia and Access Matters (IRB Number 18‐015008). The IRBs determined the study qualified for exemption from IRB review under the revised 2018 Common Rule Requirements. A waiver of HIPAA authorization was granted for accessing identifiable information from the medical records.

### 2.2 Data sources and measurement

The primary outcome measure was EHR documentation of PrEP counselling ≤6 months following the date of a qualifying STI diagnosis at an outpatient clinic visit. PrEP counselling was defined as EHR documentation of discussions and recommendations regarding PrEP based on chart review of clinical notes. Additional PrEP counselling documentation (e.g. date of counselling and provider type) was obtained by chart review. Secondary outcomes included EHR documentation of FTC/TDF prescription for PrEP and new HIV diagnoses.

An institution‐wide EHR was implemented prior to January 2014. STI and HIV testing data from the two clinics were captured in near‐real time from the medical system's STI database using a commercial business intelligence application (Qlik, Radnor, PA) [36, 50]. We performed EHR data abstraction using a standardized electronic data abstraction instrument to obtain information associated with each STI encounter collected as part of routine patient care measures. Both clinics utilized the same standardized EHR sexual and reproductive health template that included sexual history questions. Patient‐level variables included age, sex assigned at birth, gender identity (cisgender or not), gender of sexual partners, insurance status and type (insured or uninsured; public/government or commercial), primary care status (receiving primary care at the site or not) and STI test results (date and specimen source). Sexual and gender minority (SGM) youth were defined as youth who self‐reported same sex partners; assigned male sex at birth with a rectal STI diagnosis; and/or gender identity differed from sex assigned at birth. In accordance with CDC guidelines for PrEP eligibility, qualifying STI diagnoses included (1) incident *Chlamydia trachomatis* diagnosis via urine, rectal or oropharyngeal nucleic acid amplification test (NAAT) among SGM patients assigned male sex at birth; (2) incident *Neisseria gonorrhoea* diagnosis via urine, rectal, oropharyngeal or endocervical NAAT regardless of sex; and/or (3) incident syphilis diagnosis by serologic tests (i.e. Rapid Plasmin Reagin plus serum treponemal test) regardless of sex. Chlamydia was not a qualifying diagnosis for individuals assigned female sex at birth or heterosexual cisgender males [6]. Healthcare‐level variables included clinic site, being an adolescent medicine specialist (completed an adolescent medicine fellowship or not). Diagnoses in the setting of self‐reported sexual assault were excluded.

### 2.3 Statistical methods

Associations between independent categorical variables and primary care status were evaluated using Pearson's χ^2^ test or Fisher's exact test, depending on subgroup size. Associations between independent continuous variables and primary care status were evaluated using independent two‐sample *t* test or Wilcoxon rank‐sum test depending on normality of distribution. To investigate patient‐ and healthcare‐level factors associated with the primary outcome, univariable logistic regression analyses were used to calculate odds ratios (ORs) and 95% confidence intervals (CIs). Variables evaluated with univariable analysis were selected a priori based on prior research [13, 39, 43, 49, 56, 57, 58, 59]. Multivariable logistic regression analyses were used to examine the association between receipt of primary care services and primary outcome, adjusting for patient‐ and healthcare‐level factors. Due to the collinearity of rectal STI diagnosis and SGM status, two multivariable models were fit for the primary outcome: Model 1 included sex assigned at birth and SGM status; Model 2 included sex assigned at birth and rectal STI diagnosis. A sensitivity analysis was performed to examine the effect of SGM status and rectal STI diagnosis on the odds of receiving PrEP counselling, categorizing participants into four groups: SGM assigned male sex at birth with rectal STI, SGM assigned male sex at birth without rectal STI, cisgender females and cisgender heterosexual males. Adjusted odds ratios (aORs) and 95% CIs were calculated. Analyses were performed using STATA version 15.0 (College Station, TX) with two‐sided hypothesis tests and *p*‐value of <0.05 as the criteria for statistical significance.

## 3 RESULTS

### 3.1 Demographics

Over the study period, 416 HIV‐negative non‐Hispanic Black youth were diagnosed with a PrEP‐qualifying bacterial STI. Patient characteristics at first qualifying STI encounter included median age of 17 years (interquartile range (IQR) 16–18), a predominance of cisgender females with large majority covered by public/government insurance and receiving primary care services at a clinic site (Table 1). Among the 54 (13%) SGM youth, 44 were assigned male sex at birth. One transgender female patient was identified and reported only male sexual partners.

**Table 1:** Characteristics of 416 HIV‐negative U.S. non‐Hispanic Black youth diagnosed with PrEP‐qualifying bacterial STI at first STI encounter by primary care status^a^

Characteristic	Total *N* = 416	Not primary care patient (*n* = 81)	Primarycare patient (*n* = 335)	*p*
Insurance status and type				**<0.001**
Uninsured	32 (8%)	32 (40%)	0 (0%)	
Insured (public/government)	299 (72%)	37 (46%)	262 (78%)	
Insured (commercial)	85 (20%)	12 (15%)	73 (22%)	
Age at STI encounter in years, median (IQR)	17 (16–18)	17 (16–19)	17 (16–18)	**0.01**
Date of qualifying STI diagnosis in years, median (IQR)	2016 (2015–2017)	2016 (2015–2018)	2016 (2015–2017)	0.07
Sex assigned at birth, *n* (%)				**0.03**
Male	153 (37%)	21 (26%)	132 (39%)	
Female	263 (63%)	60 (74%)	203 (61%)	
Sexual and gender minority (SGM)^b^ status, *n* (%)				0.08
SGM^b^ assigned male sex at birth	44 (11%)	6 (7%)	38 (11%)	
Cisgender heterosexual male	109 (26%)	15 (19%)	94 (28%)	
Assigned female sex at birth	263 (63%)	60 (74%)	203 (61%)	
Provider training specialty, *n* (%)				0.10
Not adolescent medicine provider	323 (78%)	57 (70%)	266 (79%)	
Adolescent medicine provider	93 (22%)	24 (30%)	69 (21%)	
Clinic site, *n* (%)				0.44
Site 1	262 (63%)	48 (59%)	214 (64%)	
Site 2	154 (37%)	33 (41%)	121 (36%)	

Bold values indicate statistical significance, *p* < 0.05.

Abbreviations: IQR, interquartile range; SGM, sexual and gender minority; STI, sexually transmitted infection.

^a^ Bivariate comparisons of baseline characteristics between patients who receive primary care services versus those who do not receive primary care services were completed using *t‐*/Wilcoxon–Mann–Whitney tests for continuous variables and χ^2^/Fisher's exact tests for categorical variables. Statistical significance: *p* < 0.05, two‐tailed. Data are presented as median (IQR) for continuous measures, and *n* (%) for categorical measures.

^b^ Youth assigned male sex at birth who were diagnosed with a rectal STI were defined as SGM.

### 3.2 First qualifying STI encounter

All heterosexual cisgender males qualified for PrEP due to incident gonorrhoea diagnosis. All but one cisgender female qualified for PrEP due to incident gonorrhoea diagnosis; one qualified due to syphilis diagnosis. Although not an indication for PrEP by the most conservative CDC guidelines, concomitant chlamydia infection was common among cisgender females (124; 47%) and heterosexual cisgender males (52; 48%). Among the SGM patients assigned male sex at birth, 21 (48%) were diagnosed with an ≥1 bacterial rectal STI, and 18 (41%) had a rectal STI diagnosis with negative urine gonorrhoea and chlamydia testing.

### 3.3 Receipt of PrEP counselling

Thirty patients (7%) had documentation of PrEP counselling: 27 (90%) SGM youth assigned male sex at birth, one heterosexual cisgender male and two cisgender female youth. The median (IQR) age of patients who received counselling was 17 (IQR 15–18 years), and almost half (14; 47%) were less than 18 years old and counselled before FDA approval in adolescents. One 18‐year‐old heterosexual male primary care patient received counselling in 2018, had public insurance, was diagnosed with urogenital gonorrhoea and chlamydia, and was seen by an adolescent medicine provider. Both cisgender females received PrEP counselling in 2017, were 18–19 years old, insured and diagnosed with gonorrhoea only. They differed by primary care status, insurance type (public vs. private) and provider type (adolescent medicine vs. not). Results of univariable and multivariable logistic regressions assessing patient‐ and healthcare‐level factors and documentation of PrEP counselling are shown in Table 2. Receipt of primary care services at one of the clinic sites was not significantly associated with receipt of PrEP counselling in either Model 1 or Model 2. In both models, being diagnosed with a qualifying STI after 2016 and being assigned male sex at birth were positively associated with receipt of PrEP counselling. In Model 1, being an SGM youth and in Model 2, being diagnosed with a rectal STI were significantly associated with receipt of PrEP counselling. A sensitivity analysis (Table 3) also found markedly high odds of receiving PrEP counselling among SGM assigned male sex at birth and revealed an additive effect of being an SGM male with a rectal STI compared to SGM males without a rectal STI on the odds of receiving PrEP counselling.

**Table 2:** Univariable and multivariable logistic regression assessing patient‐ and healthcare‐level factors and PrEP counselling documentation at first qualifying encounter among 416 HIV‐negative U.S. non‐Hispanic Black youth, 2014–2019^a^

			Model 1^b^ Sex assigned at birth and SGM status		Model 2^c^ Sex assigned at birth and rectal STI	
Characteristic	Unadjusted OR (95% CI)	*p*	Multivariablea OR (95% CI)	*p*	Multivariablea OR (95% CI)	*p*
Primary care patient (Ref: Not a primary care patient)	0.96 (0.38, 2.44)	0.94	0.10 (0.01, 0.99)	0.05	0.52 (0.10, 2.77)	0.44
Insurance status (Ref: Uninsured)	2.53 (0.33,19.2)	0.37	23.8 (0.70, 806)	0.08	11.2 (0.60, 211)	0.11
Age (years)	0.93 (0.75, 1.17)	0.54	1.10 (0.74, 1.64)	0.64	0.92 (0.67, 1.27)	0.63
Year of STI diagnosis 2017–2019 (Ref: Year of STI diagnosis 2014–2016)	**2.73 (1.28, 5.83)**	**0.01**	**6.80 (1.64, 29.3)**	**<0.01**	**3.46 (1.25, 9.58)**	**0.02**
Adolescent medicine specialist provider (Ref: Not adolescent medicine)	1.83 (0.82, 4.05)	0.14	3.69 (0.86, 15.9)	0.08	1.37 (0.47, 3.99)	0.56
Clinic site #2 (Ref: Clinic site #1)	0.84 (0.38, 1.85)	0.67	2.45 (0.54, 11.0)	0.24	1.26 (0.43, 3.68)	0.67
Assigned male sex at birth (Ref: Assigned female sex at birth)	**29.2 (6.85, 125)**	**<0.001**	**26.2 (3.46, 198)**	**<0.01**	**18.6 (3.88, 89.3)**	**<0.001**
Documented same sex sexual partners (Ref: No documentation of same sex partners)	**194 (43.7, 859)**	**<0.001**	**317 (39.9, 2521)**	**<0.001**		
Diagnosed with rectal STI (Ref: No rectal diagnosis at encounter)	**54.1 (19.2, 152)**	**<0.001**			**28.0 (8.07, 97.5)**	**<0.001**

Bold values indicate statistical significance, *p* < 0.05.

Abbreviations: 95% CI, 95% confidence interval; aOR, adjusted odds ratio; Ref, Reference; SGM, sexual and gender minority; STI, sexually transmitted infection.

^a^ Univariable and multivariable logistic regression models. Statistical significance: *p* < 0.05, two‐tailed.

^b^ Variables included in Model 1: primary care status, insurance status, year of qualifying STI diagnosis, medical provider specialty, clinic site, participant age at the time of qualifying STI diagnosis, sex assigned at birth and SGM status. Youth assigned male sex at birth who were diagnosed with a rectal STI were defined as SGM.

^c^ Variables included in Model 2: primary care status, insurance status, year of qualifying STI diagnosis, medical provider specialty, clinic site, participant age at the time of qualifying STI diagnosis, sex assigned at birth and rectal STI diagnosis.

**Table 3:** Sensitivity analysis: univariable and multivariable logistic regression assessing patient‐ and healthcare‐level factors and PrEP counselling documentation at first qualifying encounter among 416 HIV‐negative U.S. non‐Hispanic Black youth, 2014–2019^a^

Characteristic	Unadjusted OR (95% CI)	*p*	Multivariable a OR (95% CI)	*p*
Primary care patient (Ref: Not a primary care patient)	0.96 (0.38, 2.44)	0.94	0.28 (0.03, 2.64)	0.27
Insurance status (Ref: Uninsured)	2.53 (0.33, 19.2)	0.38	15.7 (0.41, 597)	0.14
Aged (years)	0.93 (0.75, 1.17)	0.54	1.07 (0.71, 1.62)	0.74
Year of STI diagnosis 2017–2019 (Ref: Year of STI diagnosis 2014–2016)	**2.73 (1.28, 5.83)**	**0.01**	**9.00 (1.93, 41.9)**	**<0.01**
Adolescent medicine specialist provider (Ref: Not adolescent medicine)	1.83 (0.82, 4.05)	0.14	2.63 (0.64, 10.9)	0.18
Clinic site #2 (Ref: Clinic site #1)	0.84 (0.38, 1.85)	0.67	2.53 (0.52, 12.3)	0.25
Sexual and gender minority (SGM) status SGM^b^ assigned male sex at birth with rectal STI SGM^b^ assigned male sex at birth without rectal STI Cisgender females (Ref: Cisgender heterosexual male)	**270 (30.4, 2400)** **118 (14.0, 994)** 0.83 (0.07, 9.22)	**<0.001** **<0.001** 0.88	**939 (61.9, 14254)** **349 (29.8, 4091)** 0.60 (0.04, 8.26)	**<0.001** **<0.001** 0.70

Bold values indicate statistical significance, *p* < 0.05.

Abbreviations: 95% CI, 95% confidence interval; aOR, adjusted odds ratio; Ref, Reference; SGM, sexual and gender minority; STI, sexually transmitted infection.

^a^ Univariable and multivariable logistic regression models. Statistical significance: *p* < 0.05, two‐tailed.

^b^ Variables included in sensitivity analysis: primary care status, insurance status, year of qualifying STI diagnosis, medical provider specialty, clinic site, participant age at the time of qualifying STI diagnosis, sex assigned at birth and sexual and gender minority status.

### 3.4 Receipt of PrEP prescription

Fourteen (3%) patients were prescribed FTC/TDF for PrEP, all of whom were insured. Twelve (86%) were SGM primary care patients assigned male sex at birth. Two patients who did not receive primary care services at a clinic site were prescribed PrEP: a 17‐year‐old SGM male and a 19‐year‐old heterosexual cisgender female. The female patient prescribed PrEP was not seen by an adolescent medicine provider.

### 3.5 New HIV diagnoses

All patients who acquired HIV during our observation period were SGM male primary care patients, and none were diagnosed with HIV at the first qualifying STI encounter. The proportion of the cohort that experienced incident HIV infection was 11% (5 out of 44) among SGM patients assigned male sex at birth and 1% among the overall cohort. Three patients previously received PrEP counselling, one of whom was prescribed PrEP for 2 years but discontinued it 1 year prior to his HIV diagnosis.

## 4 DISCUSSION

We found that less than 10% of our cohort of U.S. non‐Hispanic Black youth diagnosed with an incident bacterial STI had documentation of PrEP counselling; however, the odds of receiving counselling did significantly improve over time. Contrary to our hypothesis, receipt of primary care services was not significantly associated with receipt of PrEP counselling. In 2018, non‐Hispanic Black females made up 57% of new HIV diagnoses among cisgender females in the United States [60]. Despite this alarming disparity, cisgender females in this cohort were disproportionately and significantly less likely to receive counselling than patients assigned male sex at birth. This disparity is consistent with recent data showing fewer than 5% of PrEP‐eligible cisgender females were prescribed PrEP [7]. Our results suggest that despite increased rates of PrEP counselling over time, providers did not consistently adhere to clinical practice guidelines, particularly during qualifying encounters with Black youth who were not SGM male youth. Providers’ heuristics in PrEP decision making remained unilaterally focused on sexual minority males regardless of pre‐existing primary care relationship or medical specialty. Future efforts to improve PrEP implementation may benefit from including strategies to address and/or bypass provider‐level heuristics and biases in PrEP delivery. Recent bacterial STI diagnosis is a frequently missed opportunity for medical providers to inform and counsel young Black cisgender females—a population with low rates of PrEP awareness [61, 62].

SGM patients assigned male sex at birth in this cohort represent a population particularly vulnerable to HIV acquisition. Half were diagnosed with a rectal STI and more than a third had a rectal STI with negative urine gonorrhoea and chlamydia testing. These findings underscore the importance of adhering to CDC recommendations regarding oropharyngeal, urogenital and rectal STI screening to ensure patients are appropriately tested and treated [6]. While it is encouraging that PrEP‐eligible SGM patients assigned male sex at birth were the most likely group to receive PrEP counselling, only two‐thirds received it. Our sensitivity analysis revealed an additive effect of being an SGM male with a rectal STI compared to SGM males without a rectal STI on odds of receiving PrEP counselling. Thus, rectal STI diagnosis likely influenced providers’ PrEP decision making, whereas guidelines and public health imperatives would have them discuss PrEP irrespective of this additional risk to SGM males. These findings reinforce the imperative responsibility of providers to foster an environment in which youth feel empowered to discuss sexual health goals and behaviours and engage in shared decision making about HIV prevention methods in a manner that applies clinical practice guidelines more consistently [6, 13, 25, 26, 29, 49]. The subset of the cohort that experienced incident HIV infection—all five of whom were SGM primary care patients—reflects the ongoing HIV disparities in the United States [1, 15]. Among this cohort of more than 400 PrEP‐eligible Black youth, EHR documentation of PrEP counselling was inadequate, and rates of PrEP use were rare.

Although we were unable to directly compare rates of PrEP counselling to other groups, the very low rates of PrEP counselling and prescription among this cohort are consistent with other studies that have shown inequitable PrEP care continuum outcomes among Black individuals [7, 16, 61, 63, 64] and highlight the urgent need to ensure equitable PrEP access among this priority population or risk potentiating existing HIV disparities rather than alleviating them. Further investigation is warranted to identify and remove barriers to provider‐, clinic‐ and system‐level PrEP implementation.

This study has several limitations to consider. We conducted a retrospective analysis of data collected during routine patient care measures using a standardized sexual health template that included basic sexual information (e.g. gender of sex partners) but did not include other relevant sexual risk behaviours (e.g. engagement in sex work). By employing the most conservative CDC sexual risk criteria, we likely underestimated the number of youth who could benefit from PrEP counselling, such as cisgender females with multiple chlamydia diagnoses. Therefore, the actual deficit in PrEP use is likely even more pronounced than we found. There may have been misclassification of the primary outcome if providers were providing PrEP counselling but not documenting it. However, we doubt this varied by primary care status, particularly given similar rates of documentation of same‐sex sexual partners. Moreover, we had 80% power to detect a clinically meaningful absolute difference of 10% in PrEP counselling between those not receiving primary care services versus those receiving primary care services, comparing counselling rates 3% versus 13%, which was not observed. Although our findings clearly indicate that PrEP counselling occurs primarily among SGM patients, our effect estimates were unstably large due to the rare occurrence of the outcome among cisgender females and cisgender heterosexual males. This underscores the need for large multicentre studies that enrol high numbers of cisgender heterosexual and SGM youth. Given the retrospective nature of this study, unmeasured confounding and our inability to evaluate providers’ reasons for not counselling are important concerns, including potential PrEP‐related stigma and provider bias. In addition, PrEP was not approved for all ages covered in the study for the full observational period. However, among patients who received PrEP counselling, almost half were minors counselled before U.S. FDA approval in adolescents, and neither provider specialty nor study site was associated with the odds of receiving counselling. These findings suggest that providers were aware of PrEP, and their decisions to counsel patients were not limited to concerns regarding FDA approval. Other PrEP formulations (e.g. tenofovir alafenamide/emtricitabine) were not licensed in the United States during the time of this study; therefore, we are unable to estimate the effects those changes may have on current PrEP prescribing practices. All study participants were non‐Hispanic Black youth, thus limiting the generalizability to other racial/ethnic groups. However, rates of HIV acquisition in the United States are highest among Black youth and adults, making these findings highly relevant. This study was conducted at a single urban academic institution, thus potentially limiting the generalizability to other settings. However, it is notable that counselling and prescription rates were very low at this institution despite having an STI focus, providers with expertise in the provision of sexual and reproductive healthcare for minors and early support from the department's leadership. Rates are likely lower in other settings. Future qualitative research is needed to understand the barriers to provider‐initiated PrEP counselling given the urgent need to address inequities in PrEP uptake among this U.S. priority population.

## 5 CONCLUSIONS

There is an urgent need for public health professionals and healthcare providers to overcome barriers to PrEP uptake among U.S. non‐Hispanic Black youth—a population for which HIV incidence remains disproportionately high. The results of this study suggest that receipt of primary care services—and the continuity underlying that relationship—may have little bearing on a provider's PrEP‐counselling practices for youth at increased risk for HIV acquisition. Alleviating disparate rates of PrEP access and uptake will require robust investment in PrEP‐inclusive sexual health services that are widely implemented and culturally tailored for Black youth in the United States.

## COMPETING INTERESTS

The authors declare that they have no competing interests.

## AUTHORS’ CONTRIBUTIONS

DLW, PAS, JAB, IF, SMW and RG contributed to conception or design of project. DLW, PAS, DTP, JP, JAB, SMW and RG contributed to acquisition, analysis or interpretation. DLW drafted the manuscript. DLW, PAS, JAB, SMW and RG critically revised the manuscript. DLW, PAS, DTP, JP, JAB, IF, SMW and RG gave final approval and agree to be accountable for all aspects of work ensuring integrity and accuracy.

## FUNDING

This work was supported by the National Institute of Mental Health K23MH119976 (Wood) and the Children's Hospital of Philadelphia Research Institute K‐Readiness Award (principal investigator: SMW). Research reported in this publication was supported by the National Institute of Allergy and Infectious Diseases T32AI055435 (Lautenbach). This publication was made possible through core services and support from the Penn Center for AIDS Research (P30AI045008) and Penn Mental Health AIDS Research Center (P30MH097488).

## DISCLAIMER

The content is solely the responsibility of the authors and does not necessarily represent the official views of NIH.

## Data Availability

Study data are available from the corresponding author (DLW) upon request.
